# DRC3 is an assembly adapter of the nexin-dynein regulatory complex functional components during spermatogenesis in humans and mice

**DOI:** 10.1038/s41392-022-01293-4

**Published:** 2023-01-10

**Authors:** Shushu Zhou, Shimin Yuan, Jintao Zhang, Lanlan Meng, Xin Zhang, Siyu Liu, Guangxiu Lu, Ge Lin, Mingxi Liu, Yue-Qiu Tan

**Affiliations:** 1 State Key Laboratory of Reproductive Medicine, Department of Histology and Embryology, School of Basic Medical Sciences, Nanjing Medical University, Nanjing, 211166 China; 2 Reproductive Medicine Center, Department of Obstetrics and Gynecology, the First Affiliated Hospital of Anhui Medical University, Hefei, 230022 China; 3 Clinical Research Center for Reproduction and Genetics in Hunan Province, Reproductive and Genetic Hospital of CITIC-XIANGYA, Changsha, 410008 China; 4 Institute of Reproductive and Stem Cell Engineering, NHC Key Laboratory of Human Stem Cell and Reproductive Engineering, School of Basic Medical Sciences, Central South University, Changsha, 410008 China; 5 Hunan International Scientific and Technological Cooperation base of Development and carcinogenesis, Changsha, 410008 China; 6 State Key Laboratory of Reproductive Medicine, The Affiliated Taizhou People’s Hospital of Nanjing Medical University, Taizhou School of Clinical Medicine, Nanjing Medical University, Nanjing, 211166 China

**Keywords:** Reproductive disorders, Molecular biology, Genetics

**Dear Editor**,

Male subfertility, a multifactorial disease affecting ~ 7% of the global male population, is usually caused by abnormalities in sperm flagella. The flagella and motile cilia have similar “9 + 2” axonemes and are evolutionarily conserved, being widely distributed in bacteria, archaea and eukaryotes^1^. Cilia defects also lead to primary ciliary dyskinesia (PCD), which affects approximately 1/10,000 individuals worldwide. The nexin–dynein regulatory complex (N-DRC) is a conserved structural protein complex present in the axonemes of cilia/flagella, from algae to humans. Moreover, it is composed of at least 11 components (DRC1–11) and is critical for ciliary/flagellar motility, functioning by linking the outer adjacent duplex microtubules^2^. At present, DRC1/2, crossing the long axis of the N-DRC, combined with DRC4 is considered to form a core complex that acts as a scaffold for the assembly of the “functional subunits”, namely DRC3/5-8/11.

Previous studies on mammals have revealed that defects of certain DRC components lead to various degrees of flagellar structural and/or functional damage. For example, the deficiency of DRC1, DRC7 or DRC9 causes the absence and/or disorganization of “9 + 2” axonemal arrangements, consequent to multiple morphological abnormalities of the flagella (MMAF) in humans or mice^3^. While the DRC5 defect only affects sperm motility in human and mice^4^. However, the specific function of DRC3 in mammals remains unclear.

In the *Chlamydomonas*, DRC3 was located on the flagellar axoneme as a “functional subunit” of N-DRC^2^, and mutant DRC3 leads to reduced swimming speed associated with abnormal flagellar waveform^5^. Sequence alignment showed DRC3 is highly conserved in creatures, such as *Chlamydomonas*, chicken, *Xenopus*, rat, house mouse and human.

Herein, we performed whole-exome sequencing analysis on a cohort of 314 unrelated infertile men with asthenoteratozoospermia, and identified two bi-allelic *DRC3* (NCBI: NM_031294.4) frameshift variants in two unrelated patients (M1: c.1308_1311del in II-1 in Family 1 and M2: c.1031delT in II-1 in Family 2, respectively, Fig. 1a, Supplementary Fig. S1a–d). The DRC3 mutations in the two patients were inherited from their respective parents who carried the heterozygous mutations. Spermatozoa from the patient 1 showed dramatic decrease in total and progressive motility, and displayed an MMAF phenotype (Fig. 1b, Supplementary Fig. S1e, Table S1). Patient 2 displayed obstructive azoospermia due to an iatrogenic injury to the bilateral vas deferens from childhood inguinal herniorrhaphy. Spermatozoa from percutaneous epididymal sperm aspiration of patient 2 showed an MMAF phenotype under a phase contrast microscope, but they have been used for subsequent intracytoplasmic sperm injection (ICSI) treatment. Therefore, the data was only showed from patient 1 in our study due to there were no surplus spermatozoa of patient 2 for further study. Approximately 79.4% of the cross-sections of flagellar axonemes from patient 1 displayed incomplete or disorganized microtubules, while 20.6% of the axonemes were relatively normal, with obviously a shorter N-DRC (Fig. 1c, Supplementary Fig. S1f–g). Immunofluorescence analysis of spermatozoa from patient 1 showed that DRC3, DRC5, and DRC7 were barely detectable, but DRC1, DRC2, and DRC4 levels were comparable to those observed in controls (Fig. 1d). DRC5 and DRC7 were not detected in the spermatozoa of patient 1, possibly because DRC3, DRC5, and DRC7 are all located in the N-DRC linker region, and they are relatively close in terms of their structural position^2^. Markers of basic structures, such as dynein and radial spokes, were not absent in the sperm flagella (Supplementary Fig. S1h-j).

**Fig. 1:**
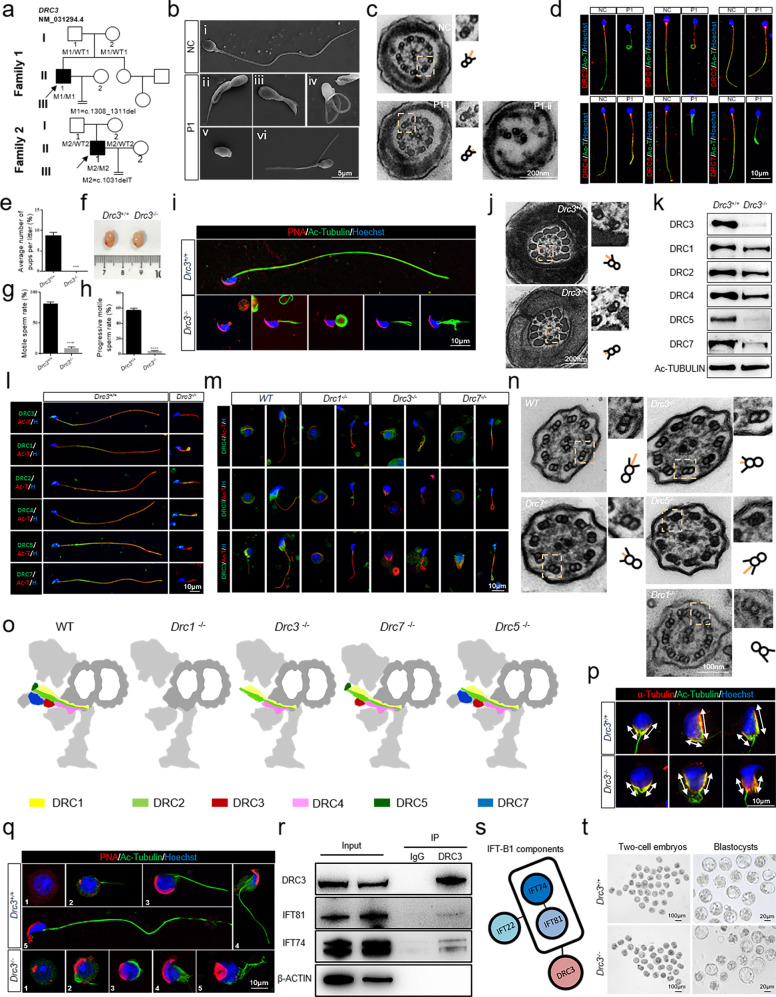
DRC3 functions as an assembly adapter for nexin–dynein regulatory complex (N-DRC) functional components during spermatogenesis in humans and mice. **a** Pedigrees of *DRC3* variants in two men with asthenoteratozoospermia. Filled and open signs indicate the affected and unaffected individuals, respectively. Arrows indicate the probands. Double horizontal lines indicate a consanguineous marriage. P1: patient1, M1: c.1308_1311del, P2: patient2, M2: c.1031delT (NCBI accession no.: NM_031294.4). **b** Scanning electron microscopy analysis of the spermatozoa obtained from a normal control (NC) and P1. Spermatozoa from the NC showed normal long flagella (i), whereas spermatozoa from P1 showed coiled flagella (ii), short flagella (iii), irregular flagella (iv), absent flagella (v), and angulation flagella (vi). Scale bar = 5 μm. **c** Transmission electron microscopy (TEM) analysis of the cross-section of spermatozoan flagella from the NC and P1. The dashed boxes mark a set of microtubular doublets and the attached accessories, including the inner and outer dynein arms and the N-DRC. Structurally complete N-DRCs were found in spermatozoan flagella from the NC, whereas the complete “9 + 2” structure with a truncated N-DRC (P1-i) (20.6%) and disordered microtubules (P1-ii) (79.4%) were detected in P1 samples. **d** Immunofluorescence analysis showed that DRC1, DRC2, and DRC4 could be detected, whereas DRC3, DRC5, and DRC7 were almost undetectable in the spermatozoa of obtained from P1 (red: DRCs, green: Ac-tubulin, blue: Hoechst). Scale bar = 10 μm. **e** An average of nine pups per litter was observed for *Drc3*^+/+^ mice, whereas *Drc3*^−/−^ male mice failed to sire any pups. (*** indicates *P* < 0.001). **f** The testis sizes were comparable between *Drc3*^+/+^ and *Drc3*^*−*/*−*^ mice at 8 weeks of age; *n* = 3. (**g**–**h**) The spermatozoa of *Drc3*^*−*/*−*^ mice showed dramatic decreases in motility and progressive motile ability compared to those from *Drc3*^+/+^ mice (*^***^P < 0.0001); *n* = 3. **i** Spermatozoa from *Drc3*^+/+^ mice showed integral, long flagella, whereas spermatozoa from *Drc*3^*−*/*−*^ mice displayed multiple flagellum abnormalities. **j** TEM analysis indicated that the N-DRC structure of the sperm flagellum axoneme from *Drc3*^*−*/*−*^ mice was obviously shorter than that from *Drc3*^+/+^ mice. The dashed boxes mark a set of microtubular doublets and the attached accessories, including the inner and outer dynein arms, and N-DRC; scale bar = 200 nm. **k**–**l** In sperm from *Drc3*^*−*/*−*^ mice, DRC3, DRC5, and DRC7 were almost undetectable, whereas DRC1, DRC2, and DRC4 levels were comparable with those in sperm from *Drc3*^+/+^ mice based on western blot analysis (**k**) and by fluorescence analysis (red: DRCs, green: Ac-tubulin, blue: Hoechst); scale bar = 10 μm (**l**). Ac-tubulin was used as the control. **m** In the spermatids from the testicular suspension, DRC4 could be detected in wild-type (WT), *Drc3* and *Drc7-*knockout mice, but was absent in *Drc1*^*−*/*−*^ mice (upper), respectively. DRC7 was absent in *Drc1*^*−*/*−*^, *Drc3*^*−*/*−*^ and *Drc7*^*−/−*^ mice (middle). DRC3 was absent in *Drc1*^*−*/*−*^ and *Drc3*^*−*/*−*^ mice, but remained in *Drc7*^*-*/-^mice (lower) (red: Ac-tubulin; green: DRC4, DRC7 and DRC3; blue; Hoechst. Scale bar = 10 μm). **n** The observation of N-DRC structures in of tracheal cilia via TEM indicated the normal and long N-DRCs in WT and *Drc5-*null mice, shorter N-DRCs in *Drc3*^*−*/*−*^ and *Drc7*^*−*/*−*^ mice, and the loss of N-DRCs in *Drc1*^*−*/*−*^ mice; scale bar = 100 nm. **o** Pattern diagrams of the N-DRC complex in WT and in *Drc1*^*−*/*−*^, *Drc3*^*−*/*−*^, *Drc7*^*−*/*−*^, and *Drc5*^*−*/*−*^mice. **p** The manchettes of *Drc3*^+/+^ mice displayed asymmetric contraction, whereas those manchettes of *Drc3*^*−*/*−*^ mice showed a symmetrical change. The two-way arrow indicates the manchette in the spermatids; scale bar = 10 μm. **q** Spermatids from a testicular suspension of *Drc3*^+/+^ mice (above) showed an extended axoneme (1–4) and an integral, long tail (5), whereas spermatids of *Drc3*^-/-^ mice (below) showed an abnormal flagellum axoneme extension (1–4) and deformed tail (5). **r** The results of immunoprecipitation in vivo revealed that DRC3 might interact with IFT81 and IFT74. **s** Pattern diagram of the interaction between DRC3 and the detected IFT-B1 components. The circle represents the DRC3 and intraflagellar transport (IFT) proteins, and the line segment represents the interactive relationship. **t** Representative two-cell embryos and blastocysts from *Drc3*^+/+^ and *Drc3*^*−*/*−*^ groups, indicating that impaired fertilization capability caused by *Drc3* dysfunction can be rescued via intracytoplasmic sperm injection (ICSI) treatment

To determine whether *DRC3* defects cause male infertility, we constructed two homozygous *Drc3*-knockout (*Drc3*^*−*/*−*^) mouse models, *Drc3*^Δ1/Δ1^ (with a 41-base pair deletion at the junction of exon 6 and the upstream intron of *Drc3* resulting in aberrant splicing and exon 6 skipping) and *Drc3*^Δ2/Δ2^ (with a deletion in exons 11–14 of *Drc3*, to simulate the frameshift variants in the patients), with the CRISPR-Cas9 system (mentioned in the Supplementary Methods). All adult *Drc3*^*−*/*−*^ mice displayed male infertility with MMAF (Fig. 1e-i, Supplementary Fig. S2–3) and without other PCD-related phenotypes (Supplementary Fig. S4, Movie S1), which reproduced the phenotypes of men with homozygous *DRC3* variants.

To understand the role of DRC3 in flagellum assembly, we then analyzed flagellum formation during spermiogenesis. The results of transmission electron microscopy indicated that the partial N-DRC was truncated in the axoneme with complete “9 + 2” structure in *Drc3*^*−*/*−*^ mice (Fig. 1j, Supplementary Fig. S5a–b), similar to that in patient 1. Immunofluorescence analysis and western blot analyses revealed that DRC5 and DRC7 were deleted in the sperm of *Drc3* knockout mice, but DRC1/2/4 was still present in the spermatozoan tail (Fig. 1k–l, Supplementary Fig. S5c–e), which was consistent with the spermatozoa of patient 1. In *Drc1*^*−*/*−*^ mice, DRC4, DRC3, and DRC7 were not assembled on the flagellar axoneme. In *Drc3*^*−*/*−*^ mice, DRC4 was still normally assembled, but DRC7 could not be detected. In contrast, in *Drc7*^*−*/*−*^mice, DRC3 could still be assembled, but DRC4 was slightly down-regulated (Fig. 1m, Supplementary Fig. S5f, S6), which was probably due to the relatively close structural location between DRC4 and DRC7^6^. DRC1/2/4 still existed in the tracheal cilia of *Drc3*^*−*/*−*^ mice (Supplementary Fig. S7), similar to that in the flagellum of *Drc3*^*−*/*−*^ mice. However, the length of the N-DRC was found to be decreased in *Drc3*^*−*/*−*^ mice, which was similar to that in to *Drc7*^*−*/*−*^ mice, but different from that in *Drc5*^*−*/*−*^ mice with a relatively intact N-DRC and *Drc1*^*−*/*−*^ mice with an almost completely absent of N-DRC (Fig. 1n). Taken together, we gave a hypothetical structural position pattern of the mammalian N-DRC (Fig. 1o), referring to the positional structural relationship in *Chlamydomonas*^2^, to show the existence of the main N-DRC components in different DRC-defect mouse models, according to our current data (Fig. 1d, k–m, and Fig. S6) and previous studies^4^. Our results suggested that DRC3 is the adapter required for “functional subunits” of the N-DRC but not “core subunits” (Fig. 1o).

Further, we observed the spermatids from testicular suspensions and found a symmetrical manchette and abnormal assembly of the axoneme during spermiogenesis in *Drc3*^*−*/*−*^ mice (Fig. 1p–q). To better understand the mechanism underlying of male infertility caused by DRC3 deficiency, we performed immunoprecipitation-mass spectrometry and co-immunoprecipitation in vivo using testicular protein from wild-type mice and co-immunoprecipitation in vitro using HEK293T cells. We found that DRC3 might interact with IFT81, IFT74 and IFT22 (Supplementary Fig. S8a–b, Fig. 1r–s). Intraflagellar transport (IFT) is vital to the axoneme assembly and structural maintenance of the cilia/flagella. IFT81, IFT74, and IFT22 participate in the formation of the IFT-B1 complex, which is critical to the axoneme protein transport^7^. In *Drc3*^*−*/*−*^ mice, IFT81, IFT74, and IFT22 were abnormally retained from early flagellum assembly to the final mature sperm (Supplementary Fig. S8c). Our results showed that DRC3 is required for flagellum assembly and normal sperm morphology and that the DRC3 defects result in retention of the IFT-B1complex, which might be related to the abnormal flagellum assembly and manchette.

To evaluate whether the infertility of *Drc3*^*−*/*−*^ male mice could be treated via assisted reproductive technology, we explored the spermatozoan fertility of *Drc3*^*−*/*−*^ male mice by performing ICSI. Our results showed that the oocytes collected from *Drc3*^+/+^ females could be fertilized with the caudal epididymal sperm from *Drc3*^*−*/*−*^ mice and that the fertilized eggs could develop into two-cell embryos and blastocysts (Fig. 1t). No statistical difference was found in the rates of two-cell embryos and blastocysts formation between *Drc3*^*−*/*−*^ male mice (59.4% and 63.2%, respectively) and *Drc3*^+/+^ male mice (51.3% and 75.0%). The percentages of live mice born via ICSI using sperm from *Drc3*^*−*/*−*^ and WT mice were 26.7% and 45.1%, respectively (Supplementary Fig. S9a–b). The results of transcriptome sequencing of blastocysts (day 5) from the two groups did not show differentially expressed genes involved in regulating sperm maturation or early embryonic development (Supplementary Fig. S9c–e, Table S2). Subsequently, ICSI treatment using the sperm extracted from the two patients with homozygous *DRC3* frameshift variants was conducted. Both couples obtained fertilized embryos and blastocysts, and the partner of patient 2 achieved a successful pregnancy and delivered a healthy baby (Supplementary Table S3). Overall, these results suggest that ICSI could be used as an efficient treatment for male infertility related to DRC3 malfunction.

Collectively, our findings indicate that mutations in *DRC3* and other DRCs are most likely an underlying cause of male infertility in a subgroup of individuals with MMAF, and reveal the differential role of DRCs during axoneme assembly. It is worth noting that this recessively inherited disease can be overcome via ICSI treatment.

## Supplementary information

DRC3 is an assembly adapter of the nexin-dynein regulatory complex functional components during spermatogenesis in humans and mice

Movie S1-a-WT

Movie S1-a-KO

Movie S1-b-WT

Movie S1-b-KO
