# Supplementary material for: DRC3 is an assembly adapter of the nexin-dynein regulatory complex functional components during spermatogenesis in humans and mice
Source: Signal Transduct Target Ther. 2023 Jan 10;8:26. doi: 10.1038/s41392-022-01293-4 (PMC9832115; doi:10.1038/s41392-022-01293-4)
Supplement: Supplementary file 1 — DRC3 is an assembly adapter of the nexin-dynein regulatory complex functional components during spermatogenesis in humans and mice [file 41392_2022_1293_MOESM1_ESM.docx]

Supplementary Materials

**DRC3 is an assembly adapter of the nexin-dynein regulatory complex functional components during spermatogenesis in humans and mice**

Shushu Zhou^#^, Shimin Yuan^#^, Jintao Zhang^#^, Lanlan Meng, Xin Zhang, Siyu Liu, Guangxiu Lu, Ge Lin, Mingxi Liu*, Yue-Qiu Tan*

Correspondence to [mingxi.liu@njmu.edu.cn](mailto:mingxi.liu@njmu.edu.cn) or [tanyueqiu@csu.edu.cn](mailto:tanyueqiu@csu.edu.cn)

**This PDF file includes:**

Graphical abstract

Supplementary Figs. S1 to S9

Supplementary Table S1, S2 and S3

Materials and Methods


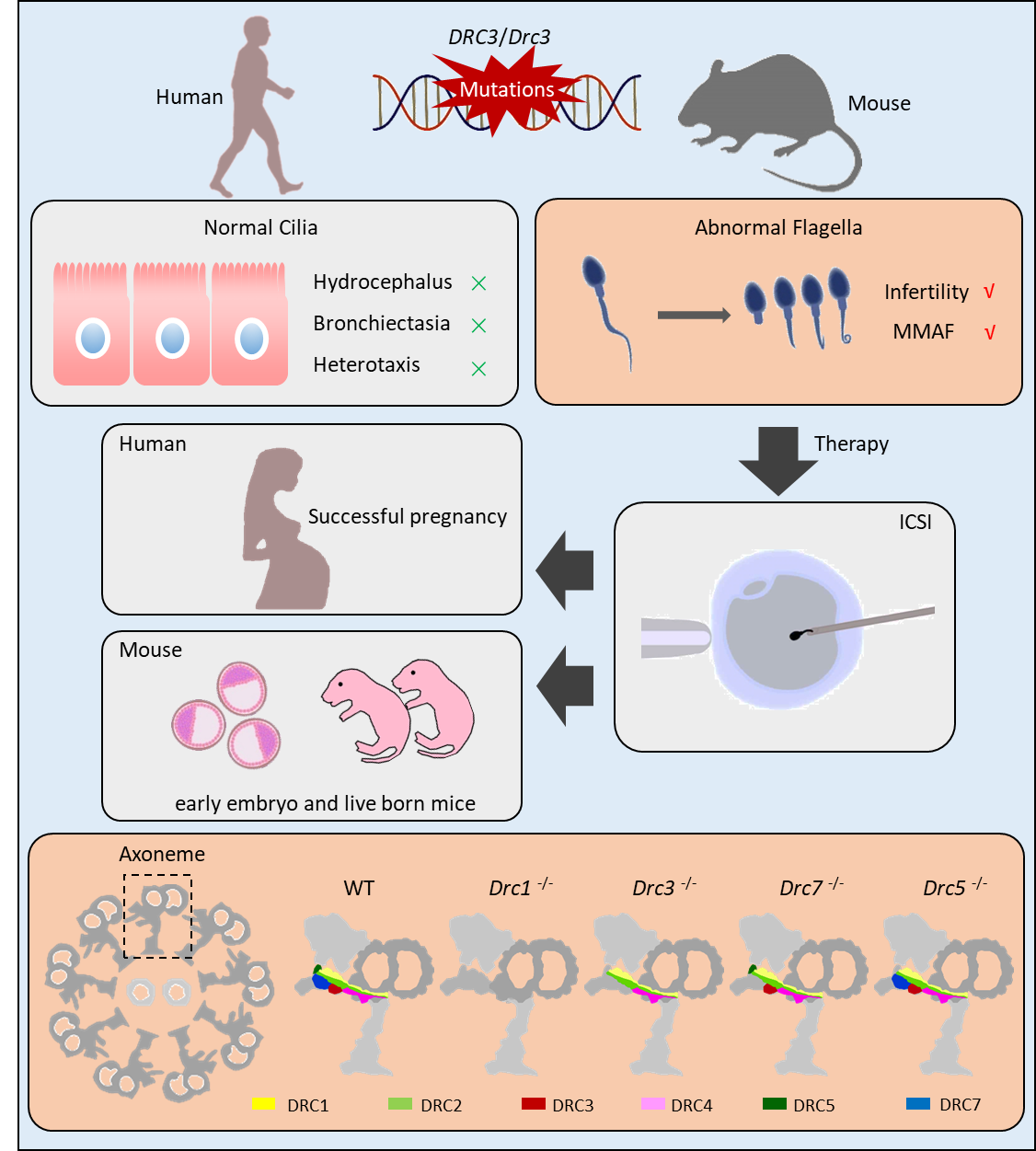


**Graphical abstract**

**
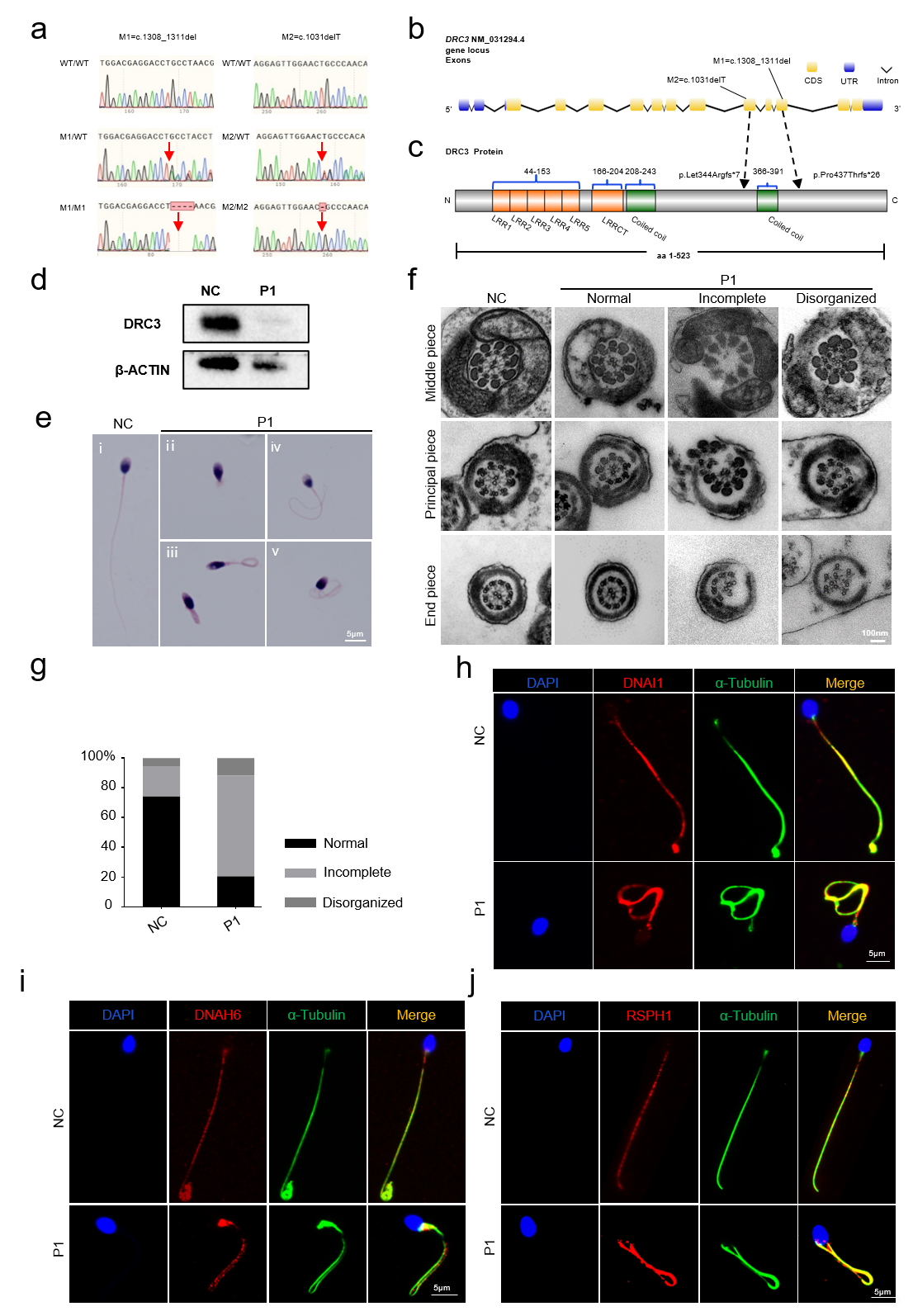
**

**Supplementary Fig. S1**.

**Phenotypic analysis of sperm in patients with homozygous *DRC3* frameshift variation.** (a) Sanger sequencing of *DRC3* variants in two men with asthenoteratozoospermia. (b-c) The two respective variants were located in exon 12 (M1) and exon 10 (M2) of *DRC3*. Both variants were frameshift variants caused by a base deletion at cDNA (NCBI accession no. NM_031294.4) nucleotide position 1308_1311 (GCCT) and 1031 (T), respectively, replacing glutamine (Pro) with threonine (Thr) at amino acid 437 and leucine (Leu) with arginine (Arg) at amino acid 344; these were both predicted to produce a truncated DRC3 protein, respectively (UniProt accession no. Q9H069). CDS, coding sequence; UTR, untranslated region; arrowheads indicate the sites of variants. (d) Western blot analysis of DRC3 in sperm proteins of normal control (NC) and Patient 1 (P1). β-ACTIN was used as the control. (e) Hematoxylin-eosin (H&E) staining of the spermatozoa obtained from a fertile NC and the man harboring a *DRC3* frameshift variant. Spermatozoa from the NC showed normal long and smooth flagella (i), whereas spermatozoa from P1 displayed typical morphological abnormalities of the flagella (MMAF) phenotypes, including absent flagella (ii), short and coiled flagella (iii), bent (iv), and irregular flagella (v) (scale bar = 5μm). (f) Ultrastructure of epididymal spermatozoa from the NC and P1 observed via transmission electron microscopy (TEM). Cross-sections of sperm flagellum showed the typical "9+2" axoneme structure in the NC, whereas three different categories of axoneme structure*s* were observed in P1, as follows: normal, incomplete, and disorganized; scale bars = 100 nm. (g) Statistical charts of different cross-sections structures of the flagellar axoneme. Total cross-section numbers for quantification in the NC and P1 were 128 and 102 respectively. (h-j) The outer dynein arms (indicated by DNAI1), inner dynein arms (indicated by DNAH6) and radial spoke (indicated by RSPH1) were retained in the sperm of P1 carrying a homozygous *DRC3* frameshift variant. (red: DNAI1/DNAH6/RSPH1, green: α-Tubulin, blue: DAPI; scale bars = 5 μm).


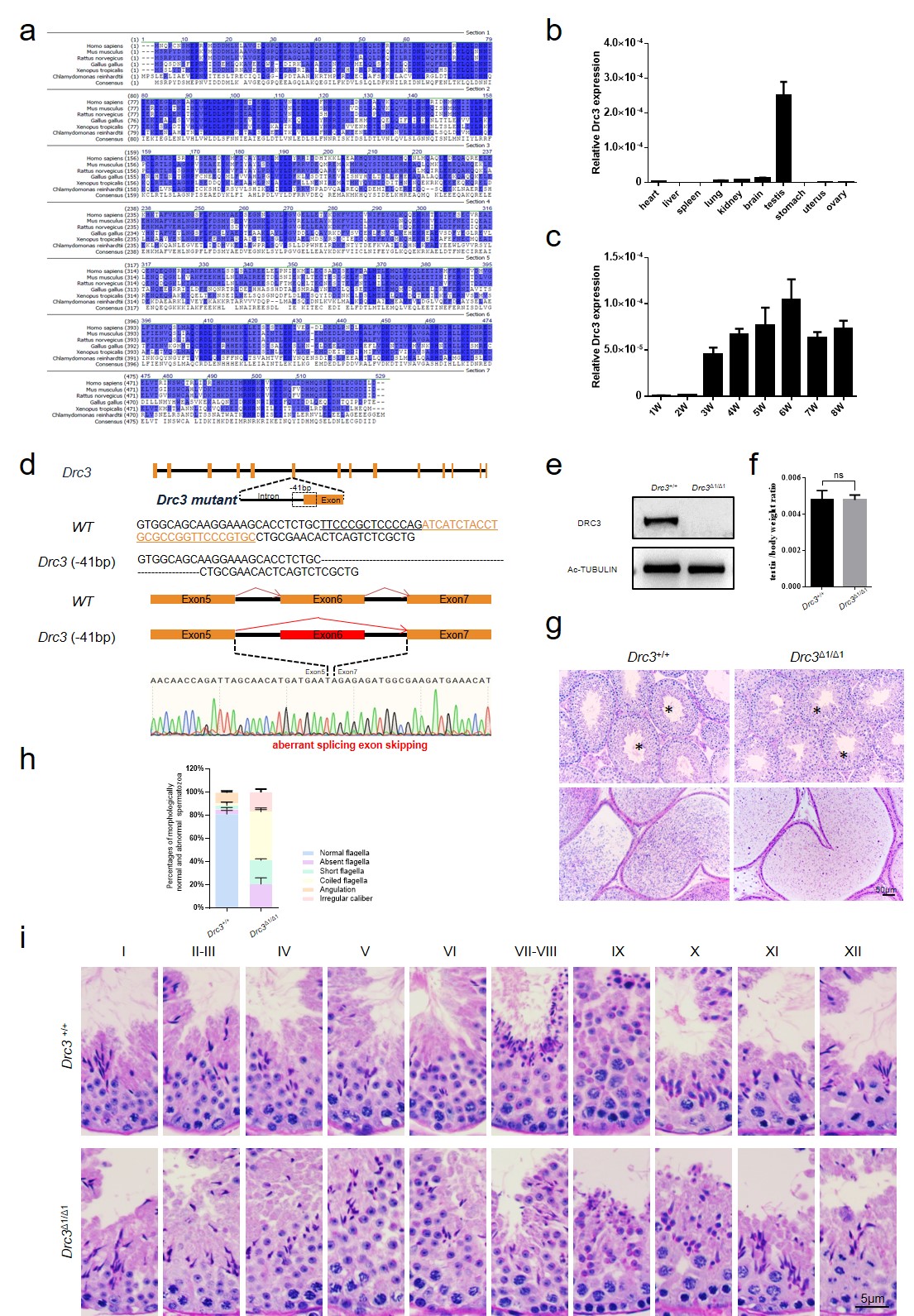


**Supplementary Fig. S2**.

**Construction of the *Drc3*^Δ1/Δ1^ mouse model and phenotypic analysis.** (a) Sequence alignment of DRC3 proteins in several metazoans (chicken, *Xenopus*, rat, house mouse, and human) and one protist (*Chlamydomonas*). (b-c) The results of RT-PCR analysis of in multiple mouse tissues and the testis at various postnatal weeks revealed that *Drc3* was highly expressed in the testis and from postnatal 3 weeks. 18S was used as a control. (d) Schematic of *Drc3* (NCBI reference sequence no. NC_000077.7) knockout mouse with a deletion of 41 bases, which are marked with a black dashed box. Black line segments represent introns and yellow rectangular boxes represent exons. Among them, 14 bases are located at the end of the intron upstream of exon 6 (underlined in black), and the other 27 bases are located at the beginning of exon 6 (underlined in yellow). Below is the RNA splicing pattern diagram, in which the broken lines with an arrow indicate the splicing pattern. The sequencing peak of *Drc3*^Δ1/Δ1^ mice is at the bottom, and the black dotted lines mark the two bases at the junction of exon 5 and 7. (e) DRC3 was undetected in sperm protein samples, based on western blot analysis, in *Drc3*^Δ1/Δ1^ mice. Ac-tubulin was used as the control. (f) Testis/body weights were comparable between *Drc3*^+/+^ and *Drc3*^Δ1/Δ1^ mice at 8 weeks of age; n=3; ns, not significant. (g) H&E staining of testis (above) and cauda epididymis (below) sections. The spermatids in the testes of *Drc3*^Δ1/Δ1^ mice were obviously deformed. The sperm in the cauda epididymis of *Drc3*^Δ1/Δ1^ mice were also significantly abnormal compared with those in *Drc3*^+/+^ mice (scale bar = 50μm). (h) Statistical analysis showing the proportion of various sperm malformations. (i) Twelve-phases analysis of spermatogenesis from testicular tissue sections of *Drc3*^+/+^ and *Drc3*^Δ1/Δ1^ mice. In both *Drc3*^+/+^ and *Drc3*^Δ1/Δ1^ mice, spermatids could be produced at all stages of spermatogenesis; however, the morphology of spermatids during the process of spermiogenesis in *Drc3*^Δ1/Δ1^ mice was obviously abnormal (scale bar = 5μm).

**
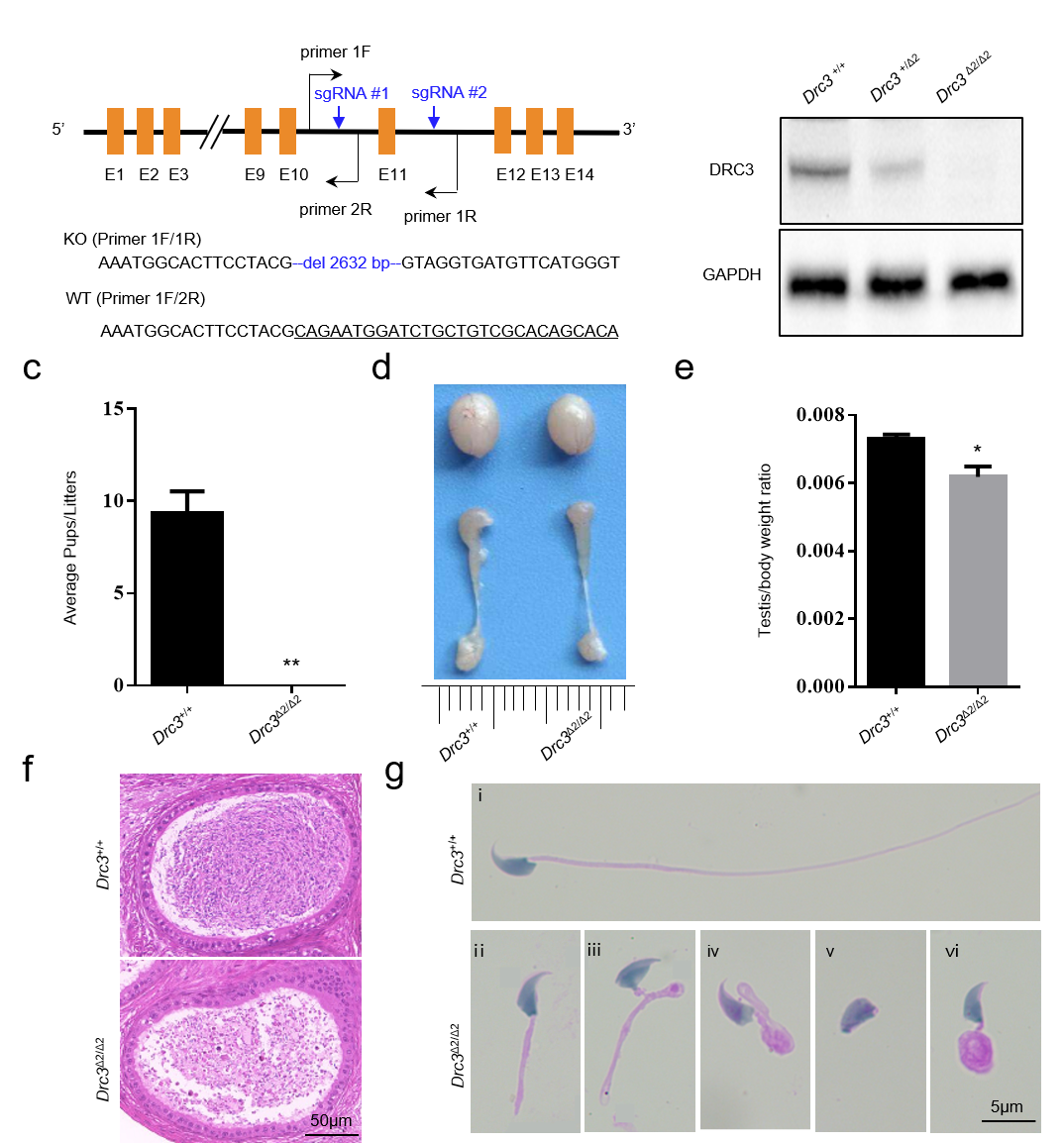
**

**Supplementary Fig. S3**.

**The *Drc3*^Δ2/Δ2^ mice carrying the simulated variant from patient 1 present with male infertility and morphological abnormalities of the flagella (MMAF).** (a) Knockout strategy to simulate the variant of patient 1 in mice. The sgRNAs were targeted in exons 11 of mouse *Drc3* to delete the partial coding region. (b) Western blotting analysis revealed almost the complete absence of DRC3 protein in the testes of *Drc3*^Δ2/Δ2^ mice. GAPDH was used as a loading control. (c) Adult *Drc3*^Δ2/Δ2^ male mice were infertile. (d) The sizes of the testis (above) and epididymis (below) were comparable between *Drc3*^Δ2/Δ2^ and *Drc3^+/+^* male mice at 8 weeks of age. (e) The testis/body weight ratio was comparable between *Drc3^+/+^* and *Drc3*^Δ2/Δ2^ mice; ns = not significant, (*P < 0.05). (f) H&E staining of epididymal sections of *Drc3^+/+^* and *Drc3*^Δ2/Δ2^ mice (scale bar = 50 μm). (g) H&E staining of the spermatozoa obtained from the cauda epididymis of *Drc3*^Δ2/Δ2^ mice displayed typical MMAF phenotypes, including short flagella (ii), angulation flagella (iii), irregular flagella (iv), absent (v), and coiled flagella (vi) (scale bar = 5μm).


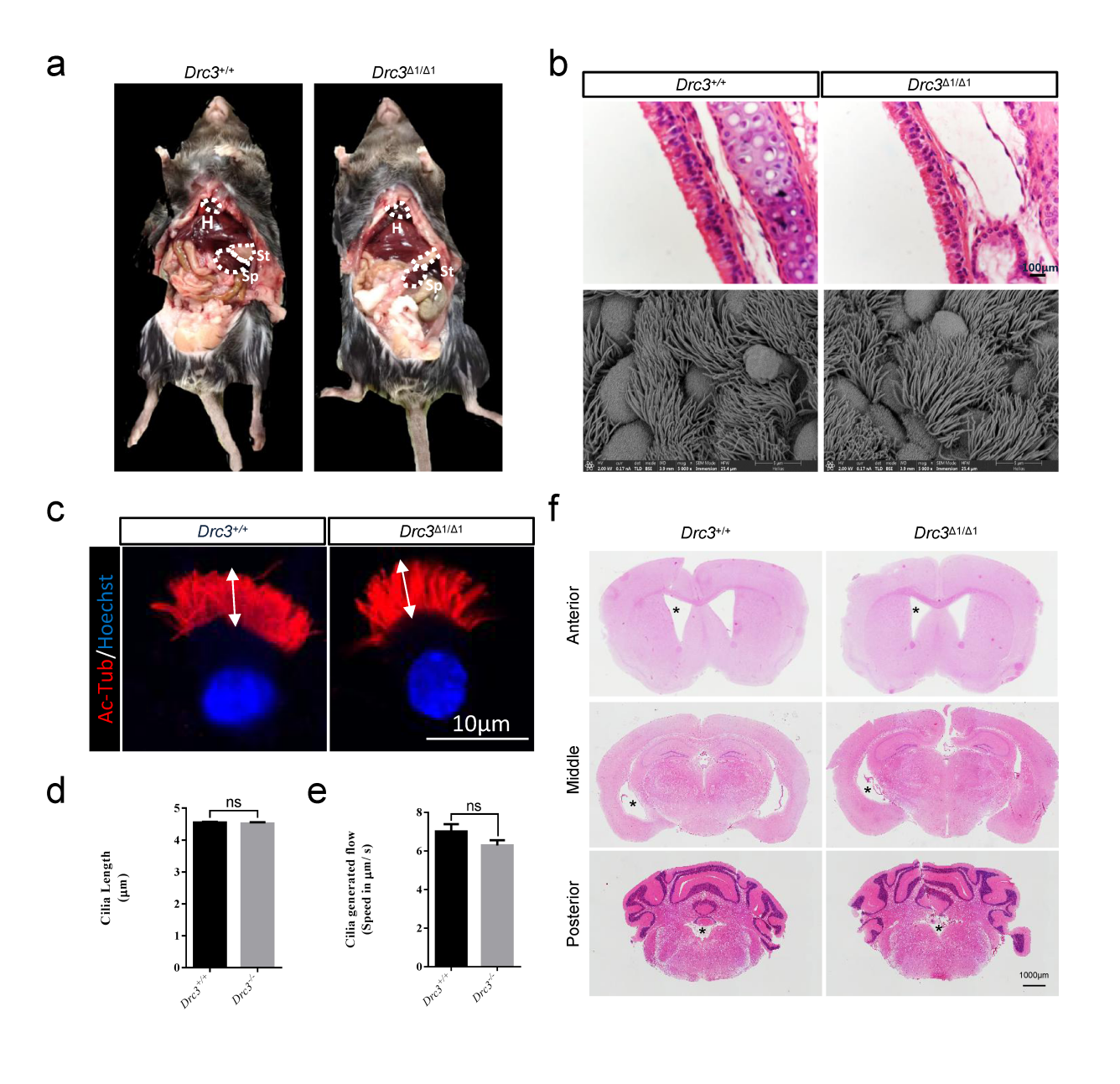


**Supplementary Fig. S4**.

***Drc3* knock-out (KO) mice do not display other primary ciliary dyskinesia (PCD) phenotypes.** (a) *Drc3*^Δ1/Δ1^ mice did not present with a laterality defect, including *situs inversus* and heterotaxy; n > 5 (H: heart, St: stomach, Sp: spleen). (b) H&E staining of tracheas of *Drc3*^Δ1/Δ1^ mice did not show mucus accumulation and the infiltration of neutrophils in the trachea (scale bar = 100μm; above), and scanning electron microscopy (SEM) showed that the morphologies of tracheal cilia were comparable between *Drc3*^Δ1/Δ1^ and *Drc3*^+/+^ mice (below; scale bar = 5μm); n = 3. (c-d) Immunofluorescence analysis of tracheal epithelial cells showed that the length of cilia (indicated by bidirectional arrow) was comparable between *Drc3*^Δ1/Δ1^and *Drc3*^+/+^ mice. Cell number = 65 and 64, respectively; scale bar = 10μm; ns indicates no significance. (e) The peripheral velocities caused by cilia swing were comparable between *Drc3*^Δ1/Δ1^ and *Drc3*^+/+^ mice; n = 3; ns indicates no significance. (f) *Drc3*^Δ1/Δ1^ mice did not display hydrocephalus in three sagittal sections including the anterior, middle, and posterior by H&E staining. Asterisks indicate the locations of the lateral ventricle (anterior and middle) and the fourth ventricle (posterior; scale bar = 500μm); n = 3.


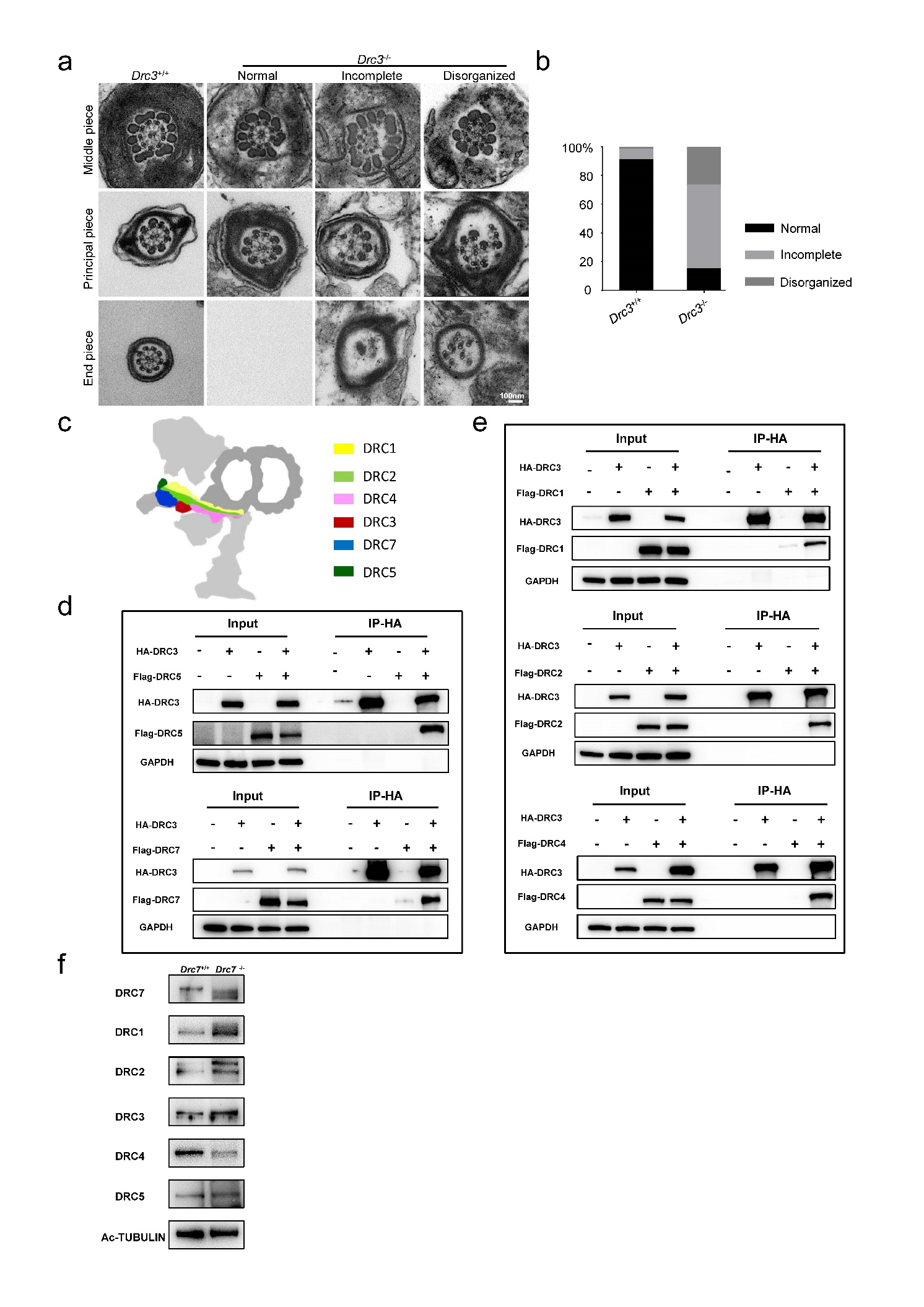


**Supplementary Fig. S5**.

**DRC3 and DRC7 dysfunction affects the assembly of the nexin–dynein regulatory complex (N-DRC) on the sperm flagellum axoneme.**

(a) Ultrastructure of epididymal spermatozoa from *Drc3* ^+/+^ and *Drc3*^−/−^ mice observed via transmission electron microscopy (TEM). Cross-sections of sperm flagella of *Drc3* ^+/+^ mice showed a typical "9+2" axoneme structure, and three different categories of axoneme structures from the middle, principal, and end piece in *Drc3*^−/−^ mice: normal, incomplete and disorganized; scale bars = 100 nm. (b) Statistical analysis of different categories of the flagellar “9 + 2” axoneme defects. Total cross-section numbers for quantification in the *Drc3*^+/+^ and *Drc3*^−/−^ mice were 71 and 103, respectively. (c) Pattern diagram of the N-DRC and DRC subunits including DRC1 (yellow), DRC2 (light green), DRC3 (red), DRC4 (violet), DRC5 (dark green), and DRC7 (blue). (d) Co-immunoprecipitation (Co-IP) experiments revealed that DRC3 interacts with DRC5 (above) and DRC7 (below) *in vitro*, using HEK293T cells. (e) Further results of Co-IP experiments revealed that DRC3 interacts with DRC1 (upper), DRC2 (middle), and DRC4 (lower) *in vitro*, using HEK293T cells. (f) Western blot analysis of N-DRC subunits showed a slightly decrease of DRC4 in sperm protein samples of *Drc7* KO mice. Ac-TUBULIN was used as a control.


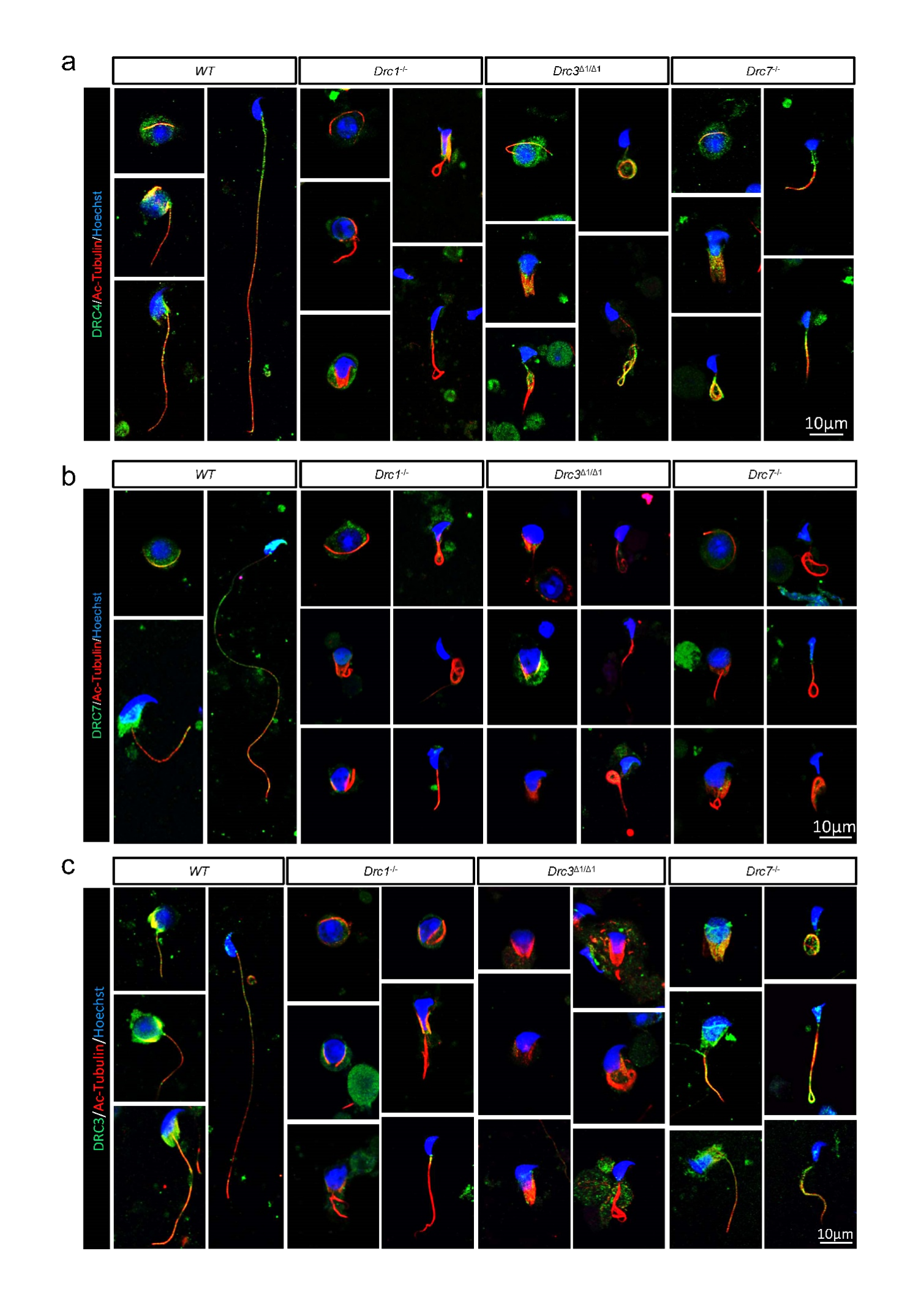


**Supplementary Fig. S6**.

**DRC3 is an assembly adapter of the nexin–dynein regulatory complex (N-DRC) functional component.** (a) In the spermatids from the testicular suspension, the core subunit of the N-DRC, DRC4, could be detected in WT mice, *Drc3*^Δ1/Δ1^ and *Drc7*-null mice, but were almost absent in *Drc1*-null mice. (b) The results of immunofluorescence staining of spermatids showed that DRC7 was absent in *Drc1*-null, Drc3-null, and *Drc7*-null mice (c) The results of immunofluorescence staining of spermatids from testicular suspensions showed that DRC3 was absent in *Drc1*-null and *Drc3*-null mice, but remained in *Drc7*-null mice (red: Ac-tubulin; green: DRC4 (a), DRC7 (b), and DRC3 (c); blue: Hoechst; scale bar = 10μm).


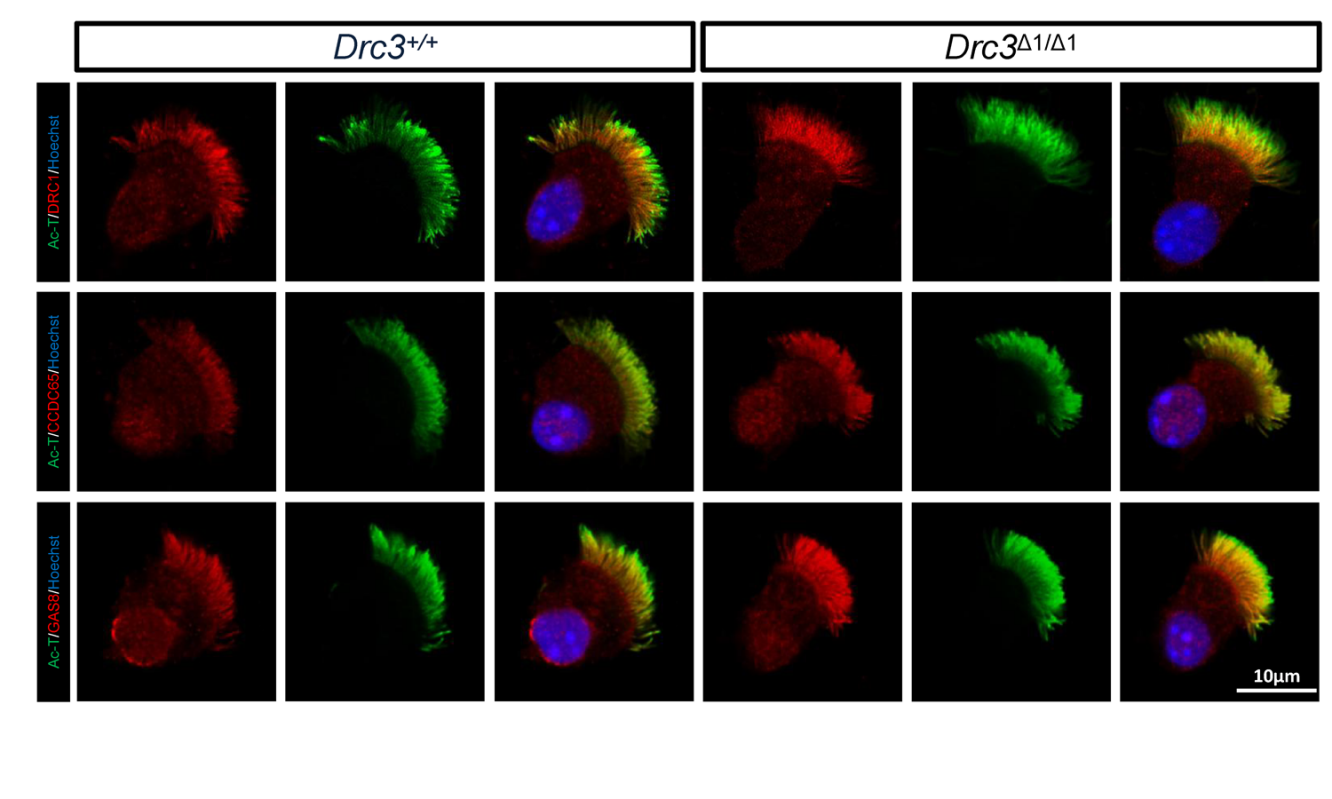


**Supplementary Fig. S7**.

**DRC3 defects do not affect the assembly of the nexin–dynein regulatory complex (N-DRC) core components in the cilia axoneme.**

Immunofluorescence analysis indicated that the expression and localization of DRC1, DRC2, and DRC4 in tracheal cilia were comparable between *Drc3*^Δ1/Δ1^ and *Drc3*^+/+^ mice (red: DRCs, green: Ac-tubulin, blue: Hoechst; scale bar = 10μm).

**
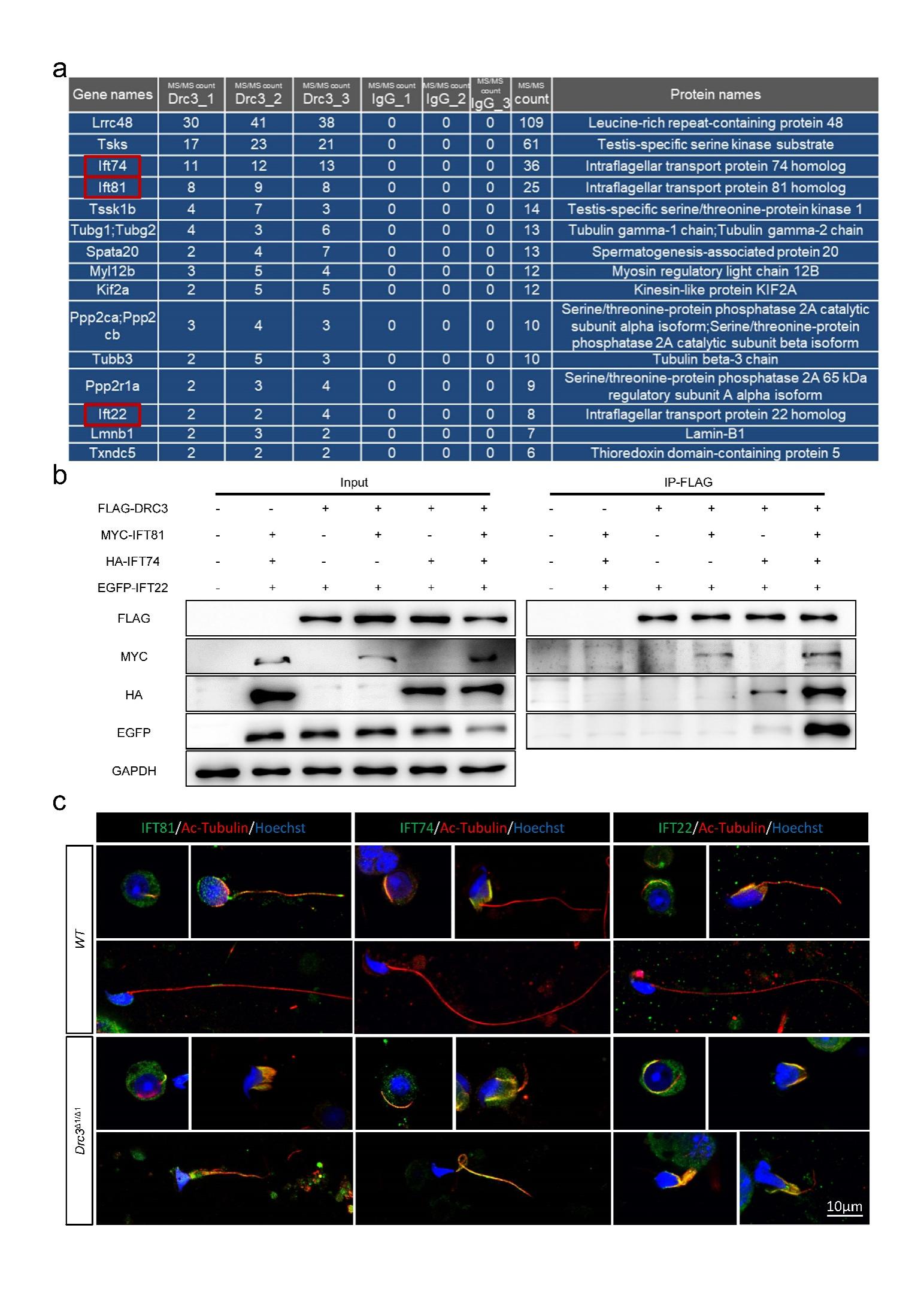
**

**Supplementary Fig. S**8

**DRC3 interacts with IFT-B1 components.** (a) Candidate proteins interacting with DCR3 by based on immunoprecipitation-mass spectrometry analysis of proteins extracted from testes of wild-type mice. A DRC3 antibody was used for the experimental group, and an IgG antibody was used for the control group. Three IFT proteins are indicated by red boxes. (b) The result of co-immunoprecipitation (Co-IP) *in vitro* showed that DRC3 interacts with IFT81/74 and that DRC3 interacts with IFT22 through IFT81/74. (c) IFT81, IFT74, and IFT22 were only presented in the manchette of spermatids in *Drc3*^+/+^ mice, whereas they could be detected in spermatids from early flagellum assembly to spermatid maturation in *Drc3*-knockout mice (red: Ac-tubulin; green: IFT81/74/22; blue: Hoechst; scale bar = 10μm).

**
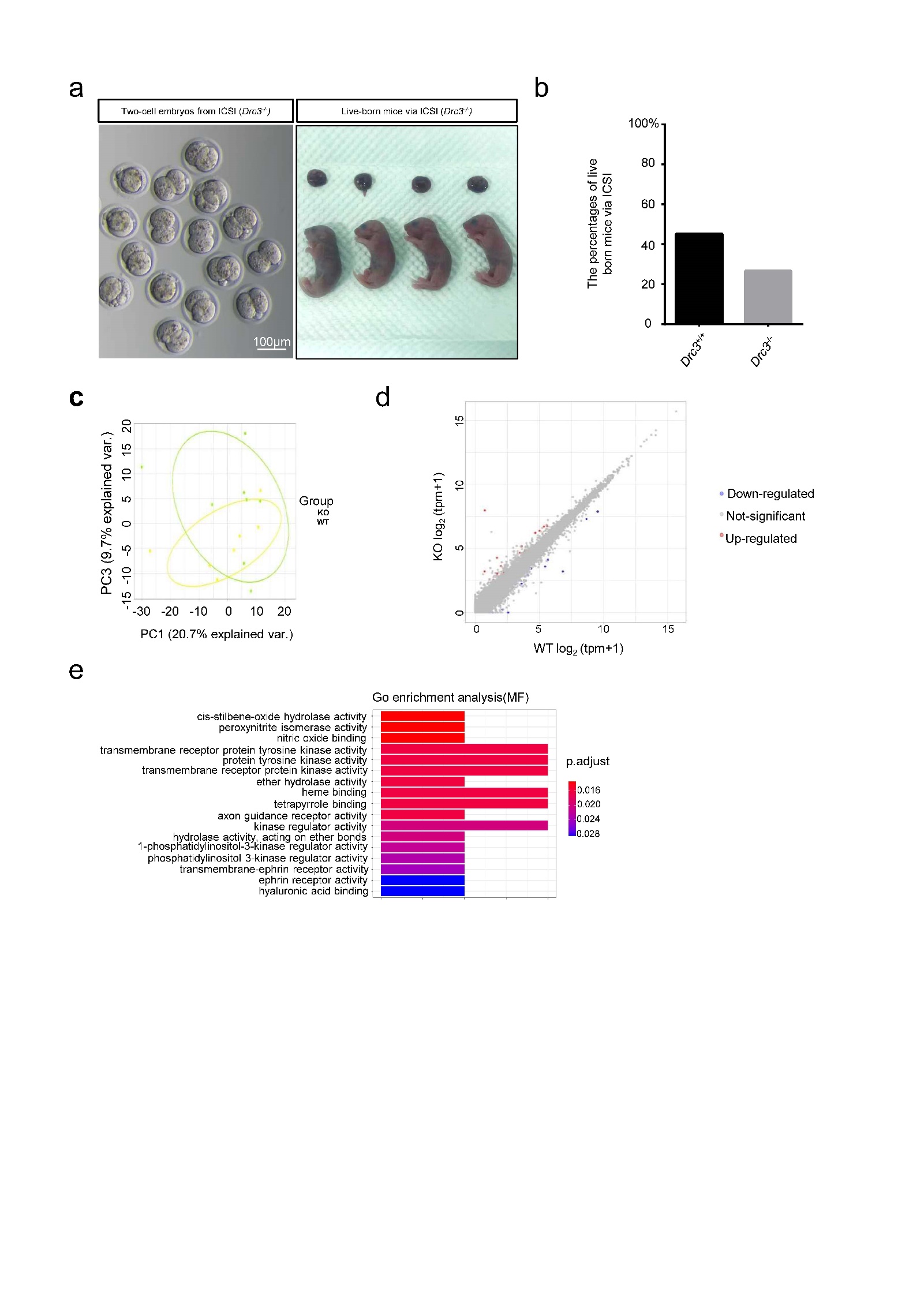
**

**Supplementary Fig. S9**

**Intracytoplasmic sperm injection (ICSI) is a feasible treatment for male infertility caused by *Drc3* deficiency** (a) Live-born mice (right) could be obtained after the transplantation (ET) of two-cell embryos from ICSI (left) using sperm of *Drc3^−^*^/^ mice. (b) The percentages of live-born mice via ICSI using sperm of wild-type (45.1%, 23/51) and *Drc3^−^*^/^*^−^* (26.7%, 4/15) mice. (c) Principal component analysis of transcriptome sequencing data of blastocysts (D5 days) from *Drc3^−^*^/^*^−^* (n=7) and wild-type male mice (n=8). (d) Scatter plot of differentially expressed genes between clusters. (e) Gene Ontology (GO) molecular function enrichment of differentially expressed genes between the two groups.

**Supplementary Table S1.**

**Table S1 Semen characteristics and sperm morphology of men harboring *DRC3* loss-of-function variants**

| **Subjects** | **Patient 1** | **Patient 2** | **Reference Values^a^** |
| --- | --- | --- | --- |
| **Semen parameters** |  |  |  |
| Semen volume(ml) | 3.2 | NA | >1.5 |
| Sperm concentration (10^6^/ml) | 70.4 | NA | >15.0 |
| Total Sperm count (10^6^) | 225.3 | NA | >39.0 |
| Motility (%) | 8.5 | NA | >40.0 |
| Progressive motility (%) | 6.3 | NA | >32.0 |
| **Sperm flagellar morphology^b^** | | | |
| Short flagella (%) | 22.36 | NA | <1.00 |
| Absent flagella (%) | 28.00 | NA | <5.00 |
| Coiled flagella (%) | 21.64 | NA | <17.00 |
| Angulation flagella (%) | 18.73 | NA | <13.00 |
| Irregular flagella (%) | 4.91 | NA | <2.00 |

a: Lower and upper reference limits according to the World Health Organization standards and the distribution ranges of morphologically abnormal spermatozoa observed in fertile individuals^1,2^. b: At least 300 spermatozoa were observed for morphology analysis. NA: Not applicable, because spermatozoa from percutaneous epididymal sperm aspiration of patient 2 who suffered from obstructive azoospermia due to iatrogenic injury of bilateral vas deferens have used for subsequent ICSI treatment. Therefore, surplus spermatozoa were unavailable.

**Table S2 Differentially expressed genes in transcriptome sequencing of blastocysts obtained from *Drc3*^-/-^ vs WT male mice sperm**

| **Gene_name** | **GeneID** | **Log2 FoldChange** | **Q-value** | **Regulated** |
| --- | --- | --- | --- | --- |
| *Vwa5a* | ENSMUSG00000023186 | 22.83 | 0.000 | upregulated |
| *Gm9376* | ENSMUSG00000092109 | 8.51 | 0.000 | upregulated |
| *H2-T10* | ENSMUSG00000079491 | 3.61 | 0.037 | upregulated |
| *Guca1a* | ENSMUSG00000023982 | 3.04 | 0.037 | upregulated |
| *Prn* | ENSMUSG00000098754 | 1.89 | 0.021 | upregulated |
| *Gm9008* | ENSMUSG00000072476 | 1.65 | 0.045 | upregulated |
| *4933434E20Rik* | ENSMUSG00000118504 | 1.62 | 0.021 | upregulated |
| *Csta2* | ENSMUSG00000095620 | 1.59 | 0.021 | upregulated |
| *Hebp1* | ENSMUSG00000042770 | 1.57 | 0.008 | upregulated |
| *Trim38* | ENSMUSG00000064140 | 1.43 | 0.021 | upregulated |
| *Arpc3* | ENSMUSG00000029465 | 1.37 | 0.000 | upregulated |
| *Cwc22* | ENSMUSG00000027014 | 1.22 | 0.034 | upregulated |
| *Ephx1* | ENSMUSG00000038776 | 1.17 | 0.021 | upregulated |
| *Ncoa7* | ENSMUSG00000039697 | 1.06 | 0.037 | upregulated |
| *Rhou* | ENSMUSG00000039960 | 1.00 | 0.037 | upregulated |
| *Thap4* | ENSMUSG00000026279 | -1.01 | 0.018 | downregulated |
| *Snapc4* | ENSMUSG00000036281 | -1.11 | 0.021 | downregulated |
| *Socs4* | ENSMUSG00000048379 | -1.15 | 0.002 | downregulated |
| *Ephb3* | ENSMUSG00000005958 | -1.23 | 0.037 | downregulated |
| *Shoc1* | ENSMUSG00000038598 | -1.27 | 0.010 | downregulated |
| *Alppl2* | ENSMUSG00000026246 | -1.39 | 0.010 | downregulated |
| *Slc6a13* | ENSMUSG00000030108 | -1.53 | 0.012 | downregulated |
| *Hapln4* | ENSMUSG00000007594 | -1.56 | 0.005 | downregulated |
| *AA467197* | ENSMUSG00000033213 | -1.67 | 0.000 | downregulated |
| *Stmn3* | ENSMUSG00000027581 | -1.95 | 0.001 | downregulated |
| *H4c4* | ENSMUSG00000061482 | -3.79 | 0.000 | downregulated |
| *Oog3* | ENSMUSG00000050810 | -4.52 | 0.030 | downregulated |
| *Nlrp5* | ENSMUSG00000015721 | -4.64 | 0.000 | downregulated |
| *Irx2* | ENSMUSG00000001504 | -5.24 | 0.007 | downregulated |
| *Otx1* | ENSMUSG00000005917 | -5.53 | 0.044 | downregulated |
| *Ccnd2* | ENSMUSG00000000184 | -5.95 | 0.037 | downregulated |
| *Fzd9* | ENSMUSG00000049551 | -6.70 | 0.028 | downregulated |
| *Phgr1* | ENSMUSG00000046804 | -6.79 | 0.000 | downregulated |
| *Nuak2* | ENSMUSG00000009772 | -7.01 | 0.044 | downregulated |
| *Gm13084* | ENSMUSG00000059218 | -7.13 | 0.000 | downregulated |
| *Tie1* | ENSMUSG00000033191 | -7.89 | 0.000 | downregulated |

**Table S3 Clinical outcomes of ICSI using the spermatozoa from men harboring homozygous *DRC3* variants**

| Subjects | Patient 1 | Patient 2 |
| --- | --- | --- |
| Male age (years) | 39 | 29 |
| Female age (years) | 40 | 36 |
| Number of ICSI cycles | 2 | 1 |
| Number of oocytes injected | 15 | 5 |
| Number (and rate) of fertilized oocytes | 13 (87%) | 5 (100%) |
| Number (and rate) of cleavage embryos | 13 (100%) | 5 (100%) |
| Number (and rate) of 8-cells | 12 (92%) | 5 (100%) |
| Number (and rate) of blastocysts | 5 (38%) | 3 (60%) |
| Number of transfer cycles | 3 | 1 |
| Clinical pregnancy rate | 0% | 100% |
| Miscarriage rate | NA | 0% |

NA: Not applicable

**Materials and Methods**

**Patients**

In total, 314 primary infertile men with asthenoteratozoospermia were enrolled from the Reproductive and Genetic Hospital of CITIC-Xiangya (Changsha, China). All individuals had normal karyotypes and no Y chromosome microdeletions. In addition, patients whose vas deferens was removed or blocked due to trauma, but for whom sperm obtained by percutaneous epididymal sperm aspiration showed immobility and severe flagellum malformations, were also enrolled. The study was approved by the ethics committees of the Reproductive and Genetic Hospital of CITIC-Xiangya. Informed consent was obtained from all participants in this study.

**Semen Characteristics Analysis and Sperm Morphological Analysis**

Semen samples of participating individuals were collected via masturbation after 3 to 7 days of sexual abstinence and analyzed after liquefaction for 30 min at 37°C. Three independent semen evaluations were performed. Semen volume, sperm concentration and motility were analyzed by the Computer-Assisted Sperm Analysis (CASA) System (Spain). Hematoxylin-eosin staining and scanning electron microscopy were using for morphological analysis of the spermatozoa. At least 300 spermatozoa of patient were counted to evaluate the percentages of morphologically abnormal spermatozoa.

**Whole-Exome Sequencing (WES) and Bioinformatic Analysis**

Genomic DNA was extracted from the peripheral blood lymphocytes of the 314 primary infertile men with asthenozoospermia using the QIAamp DNA Blood Midi Kit (Qiagen, Germany, 51106). Subsequent WES and data analyses were performed by the Beijing Genome Institute (Shenzhen, China), as described previously^3^. Briefly, the human exome was enriched using Agilent SureSelect version v.4 (Agilent Technologies, Santa Clara, CA, USA) and then sequenced with the Illumina HiSeq2500 (Illumina, San Diego, CA, USA). The obtained data were mapped to the human reference genome (UCSC hg19, <http://genome.ucsc.edu/>) with the Burrows-Wheeler Aligner software. Single nucleotide variations and short insertions and deletions were functional annotated using ANNOVAR software with OMIM, KEGG Pathway, Gene Ontology, SIFT, PolyPhen-2, MutationTaster, and the gnomAD database, 1000 Genomes Project. The pathogenicity of the candidate variants was evaluated according to the American College of Medical Genetics and Genomics (ACMG) standards and guidelines for the interpretation of variants ^4^. The candidate *DRC3* variants were further confirmed by Sanger sequencing of PCR-amplified products using specific primers (M1: Forward: 5’-CCTTGGGCCCTTAAAGTCTGT-3’; Reverse: 5’-AAAGCAGGTTAGTGTGCCCTT-3’ and M2: Forward: 5’-GCTCAAAGAGCCTCACATGTGTTC-3’; Reverse: 5’- CCATGAAGGCTACAGTTCACCT-3’).

**Plasmid**

Based on the original CAG plasmid (a gift from the Ikawa laboratory, Osaka University)^5^, the plasmids with FLAG and HA tag were constructed, respectively. The CDS sequence of *Drc3* and *Ift74* was inserted into pCAG-3xHA plasmid, the CDS sequence of *Drc1*, *Drc2*, *Drc3*, *Drc4*, *Drc5* and *Drc7* were inserted into pCAG-3xFLAG plasmid, the CDS sequence of *Ift81* was inserted into pCAG -MYC plasmid and the CDS sequence of *Ift22* was inserted into pCAG -EGFP plasmid.

**Generation of *Drc3* knockout Mice**

Two *Drc3* (Ensemble: ENSMUSG00000056598) knockout mouse strains including *Drc3*^Δ1/Δ1^ and *Drc3*^Δ2/Δ2^ were constructed. The *Drc3*^Δ1/Δ1^ mouse model was generated by CRISPR/Cas9 technology from Animal Core Facility of Nanjing Medical University using two sgRNAs targeted to knockout the exon 6 of *Drc3* (5’-CCTGCGCCGGTTCCCGTGCCTGC-3’and 5’-GCCGGAAGTCCAAGTACACGAGG -3’). Two complementary DNA oligos for each of these sgRNA targets were annealed and ligated to the BsaI-digested pUC57-T7-sgRNA vector, while sgRNA templates were amplified from sgRNA plasmids via PCR. Then, the amplified template sequences were isolated with the MinElute PCR Purification Kit (QIAGEN, Duesseldorf, Germany), and sgRNAs were generated with the MEGAshortscript Kit (Ambion, Austin, TX, USA) and purified with the MEGAclear Kit (Ambion, Austin, TX, USA) based on the instructions. Following linearization with AgeI, a Cas9 plasmid (Addgene, Watertown, MA, USA) was purified using the MinElute PCR Purification Kit (QIAGEN, Duesseldorf, Germany). And then, the mMESSAGE mMACHINE T7 Ultra Kit (Ambion, Austin, TX, USA) was used to transcribe Cas9 mRNA that was subsequently purified with the RNeasy Mini Kit (QIAGEN, Duesseldorf, Germany) based on the instructions. The Cas9 mRNA (50 ng/μL) and sgRNA (20 ng/μL) were then co-injected into murine zygotes which were transferred into pseudopregnant females. DNA was extracted with the Mouse Direct PCR Kit (Biotool, Shanghai, China). Sanger sequencing was performed for genotype identification after PCR amplification with appropriate primers (Forward: 5’-TTATAGAGGAGGAAGTCAAGG-3’ and Reverse: 5’-GAGAACAGAGCCAGGATG-3’) and Prime STAR HS DNA Polymerase (Takara, Kyoto, Japan).

For *Drc3*^Δ2/Δ2^, this mouse model was generated using the CRISPR/Cas9 technology from Cyagen Biosciences. The exon 11 was selected as the target site for knock-out (KO) mice (Fig. S3a). The sgRNA to mouse *Drc3* gene (sgRNA #1: 5’-ACAGCAGATCCATTCTGCGTAGG-3’; sgRNA#2: 5’-CCATGAACATCACCTACTTAGGG-3’) and Cas9 mRNA were co-injected into C57BL/6 mice zygote to generate a targeted line with a 2,632 bp base deletion. The founder animals were genotyped by PCR followed by sequence analysis. The specific primers (Forward/Primer 1F: 5’-CTTATGGAGAATGAGAGGTGAAC-3' and Reverse/Primer 1R: 5’-ACTACTTCTGCTATGGACTGTCT-3’) were designed for *Drc3* knockout mice, and the specific primers (Forward/Primer 1F: 5’-CTTATGGAGAATGAGAGGTGAAC-3’ and Reverse/Primer 2R: 5’-CATGGCCAGGATGAGAGCAA-3’) were used to *Drc3* WT mice.

All mice were maintained under specific-pathogen-free conditions and all animal experiments were conducted according to the protocols established by the Guide for the Care and Use of Laboratory Animals of the National Institutes of Health as well as the Institutional Animal Care and Use Committees of Nanjing Medical University (IACUC-2004020) and Central South University (CSU-2022-0001-0261).

**Tissue Collection and Histology**

Fresh testes, cauda epididymis and brain samples from WT and *Drc3* knockout adult male mice (more than five per genotype) were fixed in 4% paraformaldehyde (PFA; P1110, Solarbio) for more than 24 h and then performed for paraffin embedding. Next, fixed tissue were cut as 5 µm thickness sections and mounted on slides, and then performed with H&E staining or Periodic Acid-Schiff (PAS) Staining (G1281, Solarbio). For human and mouse spermatozoa, the human ejaculated semen and samples from cauda epididymes were washed in PBS, then fixed in 4% PFA and stained in the same way as the mice tissue.

**Western blotting**

Proteins were extracted from human sperm, mice sperm, mouse testes and culture cells using the lysis buffer (50mM Tris-HCl pH 8.2, 75Mm NaCl, 8 M urea) mixed with 50x Complete^TM^ EDTA-free Protease Inhibitor Cocktail (Roche, Basel, Switzerland) and then mixed with loading buffer, heated at 100°C for 10 min. Then the extracted protein samples were separated on SDS-PAGE, transferred onto polyvinylidene difluoride membranes, blocked with 5% skimmed milk in TBS at room temperature for 2 h, and incubated overnight at 4°C with relevant primary antibodies (Mouse anti-β-ACTIN (Proteintech, 60008-1, 1:2000), Rabbit anti-DRC3 (Abcam, ab224504, 1:500), Mouse anti-GAPDH (Abcam, ab8245, 1:2000), Mouse anti-HA-tag (MBL, M180-3, 1:2000), Mouse anti- FLAG-tag (MBL, PM020, 1:2000), self- preparing antibodies anti-DRC1 (aa 1-146), anti-CCDC65/DRC2 (aa 1-126), anti-DRC3 (aa 182-300), anti-GAS8/DRC4 (aa 1-478), anti-TCTE1/DRC5 (aa 334-498), and anti-DRC7(aa 1-292) (these anti-Murine antibodies were prepared as per prior published protocols^6^, 1:2000)), then rinsed with TBST, and followed incubated with corresponding secondary antibodies for 2 h at room temperature. The visualized of detected protein was performed with Enhanced chemiluminescence (ECL) (4600SF, Tanon, Shanghai, China).

**Fertility Testing**

Three adult *Drc3* knockout male mice (aged 8 weeks or older) in the experimental group and three adult *WT* male mice with same age. Each of them was mated with adult female mice (Male to female ratio 1:3). The next morning, the female's vaginal orifice was checked to confirm whether they mated. The successfully mated females were placed in separate cages. After 19 ~ 21 days, observed whether the female mice have offspring and counted the number of cubs in WT and KO groups.

**Sperm motility analyses**

Spermatozoa were extracted from the cauda epididymis, and dispersed in human tubal fluid (HTF, FUJIFILM Irvine Scientific, Japan) containing 10% FBS at 37°C for 5min, and then were analyzed by CASA. Hamilton Thorne’s Ceros II system (Hamilton-Thorne Research, Inc., Beverly, MA, USA) was used to dilute and analyze these samples.

**Immunofluorescence of testicular suspension, human sperm and ciliated cell**

The encapsulated testicular tissue was gently ground in phosphate-buffered saline (PBS), then centrifuged at 500 rcf for 5 min at 4°C. The ejaculated spermatozoa of patient were washed in PBS. Ciliated cells are dissociated from the inner surface of the trachea with the help of 8mm medical tracheal brushes. Testicular suspension, human spermatozoa and ciliated cells were then fixed in 4% PFA. Subsequently, the cell smears were washed with PBS three times (10 min/time), blocked with 5% BSA for 2 h, and incubated overnight at 4°C with relevant primary antibody: Mouse anti-α-Tubulin (Sigma-Aldrich, T9026, 1:500), Rabbit anti-Acetylated Tubulin: (Cell Signaling Technology,1:1000), Rabbit anti-IFT74 (Proteintech, 27334-1-AP, 1:500), Rabbit anti-IFT81 (Proteintech, 11744-1-AP, 1:500), Rabbit anti-DNALI (Sigma, HPA028305, 1:100), Rabbit anti-DNAH6 (Abcam, ab122333, 1:100), Rabbit anti-RSPH1 (Sigma, HPA017382, 1:100), Alexa Mouse anti-Acetylated Tubulin (Sigma T6793, 1:2000), Self- preparing antibodies anti-DRC1(aa 1-146), anti-CCDC65/DRC2(aa 1-126), anti-DRC3(aa 182-300), anti-GAS8/DRC4(aa 1-478), anti-TCTE1/DRC5(aa 334-498), and anti-DRC7(aa 1-292) (anti-Murine Antibody, 1:500). Following secondary antibody (Fluor 555 anti-Rabbit IgG (Yeasen, 111-165-003, 1:400),.Alexa Fluor 488 anti-Mouse IgG (Yeasen, 34106ES60, 1:400)) was mixed with Hoechst 33342 and stained at room temperature for 2 h, then washed in PBST and sealed with glycerol prior to imaging with an LSM800 confocal microscope (Carl Zeiss AG) or a TCS SP8X confocal microscope (Leica Microsystems).

**Isolation of RNA and Reverse Transcription PCR (RT-PCR)**

Total RNA was isolated and purified from multiple tissues of adult C57BL/6N mice and the testes from 1- to 8-week mice using TRIzol (15596018, Thermo Fisher Scientific, U.S.A). The reverse transcription was carried out using PrimeScript RT reagent Kit (RR047A, Takara, Japan). cDNA samples were then diluted according to 1:4 and analyzed via qPCR in a mixed system containing appropriate forward/reverse primers (Forward/Primer F :5’- CGTCTACTGCAAGGGGAGAC-3' and Reverse/Primer R:5’- CTCCTTCACATACGGTTCCTGA-3’), cDNA, and AceQ qPCR SYBR Green Master Mix (Vazyme, Q131, Nanjing, China). The 18s rRNA (Forward/Primer F :5’- CATTCGAACGTCTGCCCTATC-3' and Reverse/Primer R:5’- CCTGCTGCCTTCCTTGGA-3’) was utilized as a normalization control. To verify the expression of *Drc3*^Δ1/Δ1^ mice, we also extracted adult WT and *Drc3*^Δ1/Δ1^ mouse testicular RNA and obtained cDNA according to the above method. The fragment was amplified via PCR by using the forward primer spanning exon 4 and exon 5 and the reverse primer locating on exon 8 (Forward/Primer F :5’- GGCAACAACCAGATTAGCAACA-3' and Reverse/Primer R:5’- CCTCCACATCCTCGGAATACAT-3’). The complete loss of exon 6 was confirmed by sequencing.

**Co-immunoprecipitation (Co-IP)**

Proteins were extracted from HEK293T cells transfected with overexpression plasmids. Protein A magnetic beads were used to remove the non-specific binding protein. After the magnetic beads were removed, 50μl sample was used as input, and primary anti-HA or FLAG antibody were added to the remaining protein samples and incubated overnight at 4°C. The next day, samples were mixed with 50μl Protein A magnetic beads for 2 h at 4°C, then washed with PBST. After the magnetic beads were removed, the IP pellets and extract samples were boiled and used for WB.

**Mass spectrometry**

Self-preparing DRC3 antibody and IgG antibody were incubated with proteins extracted from testis of wild-type mice for immunoprecipitation, respectively. Eluates were precipitated with five volumes of -20°C pre-chilled acetone followed by trypsin digestion. Trypsin peptide mixture was loaded into the analytical column (Acclaim PepMap C18, 75 μm x 25 cm; Thermo Scientific). The eluted peptides were separated by linear gradient before subsequent analysis. LC-MS/MS analysis was performed on EASY-nanoLC 1000 system (Thermo Scientific) coupled to an Orbitrap Fusion Tribrid mass spectrometer (Thermo Scientific) by a nano spray ion source. The raw data is accessible from the ProteomeXchange Consortium via the dataset identifier PXD033074.

**Transmission electron microscopy (TEM)**

Ultrastructural analyses of human and mouse spermatozoa using TEM were conducted as previously described^7-9^. To assess microstructural anatomy, tracheal and epididymal specimen were fixed in 2.5% glutaraldehyde, with subsequent exposure to 2% OsO4, and then Araldite for embedding purposes. Ultrathin 80 nm sections staining were done with uranyl acetate and lead citrate, and analysis with an electron microscope (JEM.1010, JEOL). Cross sections of more than 30 spermatozoa from 3 discontinuous sections were observed in each sample. The N-DRC structure can always be found in the cross sections with clear microtubules and RS.

**Analysis of cilia length in multiciliated cells and cilia-generated flow in mouse trachea**

Ciliated columnar epithelial cells on tracheas from adult WT and *Drc3* KO male mice (n=3 for each) were isolated and stained using anti-acetylated α-tubulin antibody and Hoechst as described above. The fluorescence images photographed were performed with the confocal microscope (Leica TCS SP8AOBS) and the cilia length was determined using LAS X (Leica) software by measuring the length of the ciliary tuft of each cell at three locations and averaging the measurements. At least twenty cells were analyzed in each experimental group.

Fresh tracheas from10-day mice were opened on the dorsal side and added to high-glucose DMEM containing 10% FBS (Gibco) and 10 mM DTT (Sangon). The tissues were cut as ~5 mm wide fragments and then transferred to a confocal dish (BDD012035, BIOFIL) and a scotch tape spacer was used to facilitate their imaging under a 40× objective (CFI S Plan Flour ELWD NAMC) with an inverted microscope (Eclipse Ti2-U, Nikon). Movies were recorded for 10 s at frame rates of 25 fps.

To analyze cilia-generated flow in mouse trachea. Beads (1:200, Thermo, FluoSpheres Fluorescent Microspheres 1 micron) were added to the trachea explants and the bead flow was recorded at 23 fps for 6 s using fluorescent microscopy at 24-27°C. Trajectories of fluorescent microspheres near the ciliary tuft were analyzed using the 26 ImageJ (ImageJ 1.53c, USA).

**Scanning electron microscopy (SEM)**

Fresh tracheas of mice were fixed for 2 h with 2.5% phosphate-buffered glutaraldehyde at 4°C. Tracheas were then allowed to attach to coverslips coated with poly-L-lysine. Both sample types were then washed with PBS, dehydrated with a chilled ethanol gradient (30%, 50%, 70%, 80%, 90%, and 100%), and subjected to critical point drying with a Lecia EM CPD300 Critical Point Dryer (Wetzlar, Germany). Samples were then attached to appropriate specimen holders and coated with gold particles via the use of an ion sputter coater (EM ACE200, Leica). The images photographed were performed with the Helios G4 CX scanning electron microscope (Thermo Scientific).

**ICSI treatment of *Drc3*^Δ2/Δ2^ mice**

The preparation of mature oocyte and sperm and in vitro injection process were performed according to methods described previously^10^. Briefly, mature oocytes were collected from B6D2F1 female mice (8 to 12 weeks old) after superovulated treatment by injection of 10 IU of pregnant mare serum gonadotropin (PMSG, Livzon), followed by injection of 10 IU of human chorionic gonadotropin (hCG, Livzon) 48 h later. After 15–16 h, cumulus-intact oocytes were harvested and umulus cells were removed by hyaluronidase (FUJIFILM Wako Pure Chemical) treatment. The sperm head of wild-type mice and the whole sperm of *Drc3*^Δ2/Δ2^ mice was injected separately into a mature oocyte by a piezo manipulator (PrimeTech, Japan). Then, the formation of two cell and blastocyst were observed at 24 and 96 hours, respectively.

**Transcriptome sequencing of blastocysts**

Mouse blastocysts (day 5) obtained through ICSI were prepared by identical protocols. Single blastocyst was washed twice with 1× PBS containing 0.1% BSA before placing in lysis buffer by gentle mouth pipetting. RNA was isolated from single blastcyst and RNA-seq libraries were prepared using the SMARTSeq2 protocol (PMID: 24385147, 31588047). Briefly, following cell lysis, poly-A(+) RNA was reverse transcribed using SuperScript II reverse transcriptase (Thermo Fisher Scientific, Cat#18064071) with a strand-switch reaction to add a reverse primer (TSO: 5’-AAGCAGTGGTATCAACGCAGAGTAC/rGrG+G-3’, where “r” indicates a ribonucleic acid base and “+” indicates a locked nucleic acid base, Exiqon, Cat#500100) for second-strand synthesis. The cDNA was amplified by PCR (18 cycles, ISPCR primer: 5’-AAGCAGTGGTATCAACGCAGAGTAC-3’, Exiqon) using KAPA HiFi Hot-Start ReadyMix (KAPA Biosystems, Cat#KK2602) and the following thermal cycle conditions: 98°C (3 min); followed by 18 cycles of 98℃ (15 s), 67℃ (20 s), 72℃ (6 min); and a final elongation step 72℃ (5 min). Following amplification, the PCR products were purified using Ampure XP beads (Beckman Coulter, Cat#A63881). The Agilent 2100 Bioanalyzer (Agilent Technologies) was used to inspected the fragment size and yield of the product of each sample, and single-cell libraries were constructed using the Nextera XT DNA Library Preparation Kit (Illumina, Cat#FC-131-1096) following manufacture’s instructions. All libraries were amplified to make DNA nanoball (DNB) and sequenced on DNBSEQ Platform (BGI-Shenzhen, China). All reads passed filter were trimmed to remove low-quality bases and adaptor sequences using fastp (v0.20.0). Reads were aligned to the mouse reference genome of GRCm38 (without masking repeats) using STAR (v2.7.3a), and Transcripts per million (TPM) were calculated and normalized using StringTie (v2.0.4). Differentially expressed genes were analysed using R package DESeq2 (filtered with qvalue ≤ 0.01 and Foldchange ≥ 2). PCA and scatter plot using R package ggplot2. Differentially expressed genes were assigned to the GO terms (http://www. geneontology.org/).

**Intracytoplasmic Sperm Injection (ICSI) treatment of *DRC3* deficient patients**

ICSI were performed in the two patients as follows: the cumulus cells of oocyte–cumulus complex were removed by briefly rinsing in 80 IU/mL [hyaluronidase](https://www.sciencedirect.com/topics/biochemistry-genetics-and-molecular-biology/hyaluronidase) and gentle aspiration. Denuded oocytes were washed and transferred into G-MOPS medium (Vitrolife, 10130) and the matured oocytes were selected to injected. Sperm from ejaculated semen of Patient 1 and obtained by percutaneous epididymal sperm aspiration of Patient 2 were selected for injection, on the basis of whether they had relatively good morphology and motility and were then immobilized in polyvinylpyrrolidone before injection. The oocyte was held by the holding pipette, and the microinjection needle was pushed through the zona and into the cytoplasm. A small amount of cytoplasm was sucked into the microinjection needle and released back into the oocyte along with the sperm. The injected oocyte was released from the holding pipette and was transferred into G-IVF medium and cultured in 6% CO2, 5% O2, and 89% N2 at 37°C in a humidified incubator. The written informed consents were signed by each couple that accepted ICSI treatment.

**Statistical analysis**

Data are given as mean ± SEM and were compared via two-tailed unpaired Student’s

t-test. *P < 0.05, ** P < 0.01, *** P < 0.001, **** P < 0.0001. Microsoft Excel and GraphPad Prism 6.0 were utilized for all statistical testing.

**References**

1 Cooper, T. G. *et al.* World Health Organization reference values for human semen characteristics. *Hum Reprod Update* **16**, 231-245, doi:10.1093/humupd/dmp048 (2010).

2 Auger, J., Jouannet, P. & Eustache, F. Another look at human sperm morphology. *Hum Reprod* **31**, 10-23, doi:10.1093/humrep/dev251 (2016).

3 Tan, Y. Q. *et al.* Loss-of-function mutations in TDRD7 lead to a rare novel syndrome combining congenital cataract and nonobstructive azoospermia in humans. *Genet Med* **21**, 1209-1217, doi:10.1038/gim.2017.130 (2019).

4 Richards, S. *et al.* Standards and guidelines for the interpretation of sequence variants: a joint consensus recommendation of the American College of Medical Genetics and Genomics and the Association for Molecular Pathology. *Genet Med* **17**, 405-424, doi:10.1038/gim.2015.30 (2015).

5 Hasuwa, H. *et al.* Transgenic mouse sperm that have green acrosome and red mitochondria allow visualization of sperm and their acrosome reaction in vivo. *Exp Anim* **59**, 105-107, doi:10.1538/expanim.59.105 (2010).

6 Liu, M. *et al.* SHCBP1L, a conserved protein in mammals, is predominantly expressed in male germ cells and maintains spindle stability during meiosis in testis. *Mol Hum Reprod* **20**, 463-475, doi:10.1093/molehr/gau014 (2014).

7 Zhang, J. *et al.* Loss of DRC1 function leads to multiple morphological abnormalities of the sperm flagella and male infertility in human and mouse. *Hum Mol Genet* **30**, 1996-2011, doi:10.1093/hmg/ddab171 (2021).

8 Castaneda, J. M. *et al.* TCTE1 is a conserved component of the dynein regulatory complex and is required for motility and metabolism in mouse spermatozoa. *Proc Natl Acad Sci U S A* **114**, E5370-e5378, doi:10.1073/pnas.1621279114 (2017).

9 Wirschell, M. *et al.* The nexin-dynein regulatory complex subunit DRC1 is essential for motile cilia function in algae and humans. *Nat Genet* **45**, 262-268, doi:10.1038/ng.2533 (2013).

10 Liu, C. *et al.* Bi-allelic DNAH8 Variants Lead to Multiple Morphological Abnormalities of the Sperm Flagella and Primary Male Infertility. *Am J Hum Genet* **107**, 330-341, doi:10.1016/j.ajhg.2020.06.004 (2020).
